# The effect of stem cell factor on proliferation of human endometrial CD146^+^ cells

**Published:** 2016-07

**Authors:** Mehri Fayazi, Mojdeh Salehnia, Saeideh Ziaei

**Affiliations:** 1 *Department of Medical Sciences, Najafabad Branch, Islamic Azad University, Najafabad, Iran.*; 2 *Department of Anatomy, Faculty of Medical Sciences, Tarbiat Modares University, Tehran, Iran.*; 3 *Department of Midwifery, Faculty of Medical Sciences Tarbiat Modares University, Tehran, Iran.*

**Keywords:** *Endometrium*, *Stem cell factor*, *Stromal cells*

## Abstract

**Background:**

Stem cell factor (SCF) is a transcriptional factor which plays crucial roles in normal proliferation, differentiation and survival in a range of stem cells.

**Objective:**

The aim of the present study was to examine the proliferation effect of different concentrations of SCF on expansion of human endometrial CD146^+^ cells.

**Materials and Methods:**

In this experimental study, total populations of isolated human endometrial suspensions after fourth passage were isolated by magnetic activated cell sorting (MACS) into CD146^+^ cells. Human endometrial CD146^+^ cells were karyotyped and tested for the effect of SCF on proliferation of CD146^+^ cells, then different concentrations of 0, 12.5, 25, 50 and 100 ng/ml was carried out and mitogens-stimulated endometrial CD146^+^ cells proliferation was assessed by MTT assay.

**Results:**

Chromosomal analysis showed a normal metaphase spread and 46XX karyotype. The proliferation rate of endometrial CD146^+^ cells in the presence of 0, 12.5, 25, 50 and 100 ng/ml SCF were 0.945±0.094, 0.962±0.151, 0.988±0.028, 1.679±0.012 and 1.129±0.145 respectively. There was a significant increase in stem/ stromal cell proliferation following in vitro treatment by 50 ng/ml than other concentrations of SCF (p=0.01).

**Conclusion:**

The present study suggests that SCF could have effect on the proliferation and cell survival of human endometrial CD146^+^ cells and it has important implications for medical sciences and cell therapies.

## Introduction

Human endometrium is structurally and functionally divided into functionalis and basalis layers (1, 2). The presence of some stem cell populations in human endometrium was shown before and these cells are considered to participate in regeneration and remodeling of endometrium during the menstrual cycles (3-6). Endometrial regeneration also occurs after each endometrial incision and pregnancy (7, 8). Recent studies have reported that endometrial stem cells have mesenchymal stem cell (MSCs) properties such as self-renewal, high proliferative potential and differentiation into chondrogenic, adipogenic, osteogenic and neurogenic lineages (5, 9, 10).

The unique CD marker for endometrial stem cells was not shown so far (7). CD146 marker has been used to isolate and identify human endometrial mesenchymal stem cells (EnMSCs) (9, 11-14). The stem cells within the endometrium have a very limited population thus it needs to have focused some attempts to establish an in vitro culture condition to expand the number of stem cells (1). Stem cell factor (SCF) is a transcriptional factor which plays crucial roles in normal proliferation, differentiation and survival in a range of cell types (15, 16). Binding of SCF to its receptor c-Kit can trigger several intracellular signaling pathways (17-20). It has been shown that SCF treatment of stem cells induces activation of phosphatidylinositol-3-kinase (PI3K)-Akt signaling that promote cell survival and proliferation (19, 20). It also protects the cells from oxidative stress by activating its receptor c-Kit (15). Also this factor is essential for survival and proliferation of germ cells and their migration toward the gonads (21). However there are some reports for characterization of endometrial stem cells but little is known about the possible effect of SCF on proliferation of endometrial stem cells especially human endometrial CD146^+^ cells.

The aim of the present study was to examine the proliferation effect of different concentrations of SCF on the expansion of human endometrial CD146^+^ cells.

## Materials and methods

Reagents and materials of this research were obtained from Invitrogen (Manchester, UK) except mentioned otherwise. Ethics approval was obtained from the ethics committee of medical faculty of Tarbiat Modares University (Ref no= 52/11224). Informed written consent was obtained from each woman.

**Human endometrial samples**

Human endometrial tissue was randomly obtained from women at proliferative phase aged 30-45 yrs (n=10) undergoing hysteroscopy for non-endometrial pathologic conditions in Atieh hospital (Tehran, Iran) as an experimental study. These women had not taken exogenous hormones for 3 months prior to surgery. The normality of endometrial tissue was proved by histological examination according to well-established histological criteria for the normal menstrual cycle and confirmed by experienced histopathologist.

**Preparations of human endometrial cells**

Preparation of human endometrial cells was done according to the method described earlier by Chan *et al* in the laboratory of Tarbiat Modares University (3). Briefly, human endometrial tissue was scraped from the myometrium and washed in phosphate buffer saline (PBS). Then the tissues were minced in medium containing Dulbecco Modified Eagle Medium/Hams F-12 (DMEM/F-12) supplemented with 100 mg/ml penicillin G sodium, 100 mg/ml streptomycin sulphate B and 10% fetal bovine serum (FBS). 

Then tissues fragments were digested and dissociated into single-cell using collagenase type III (300 µg/ml; Sigma, Munich, Germany), deoxy ribonuclease type I (40 µg/ml; Sigma, Munich, Germany) and mechanical methods for 60-90 min. Then to remove glandular and epithelial components, they were pipetted and passed through meshes of 150, 100, 40 sieve (BD Biosciences, Erembodegem, Belgium) respectively (22). 

**Culture of human endometrial cells**

Purified EnMSCs were seeded using DMEM/F-12 containing antibiotics and 10% FBS that incubated at 37^o^C in 5% CO_2_. The media were changed every 2-3 days. The cells were passaged when cultures reached 80-100% of confluency. For passaging, after washing with PBS, the cells were treated with 0.05% trypsin and 0.02% ethylene diamine tetra acetic acid (EDTA) at 37^o^C for 3 min and then complete DMEM added to stop the enzyme reactions. Afterward, they were centrifuged and cultured in culture media. 

**Cell isolation by magnetic-activated cell sorting (MACS)**

Total populations of isolated human endometrial suspensions (up to 1×10^7^ cells/100 µl) after fourth passage were enzymatically dissociated into a single cell suspension. The cells were incubated with rabbit anti-human CD146 monoclonal antibody (1:200; Abcam, Cambridge, UK) at 37^o^C for 45 min. After washing the cells with PBS, they were incubated with anti-rabbit IgG MicroBeads antibody (Miltenyi Biotec, Bergisch Gladbach, Germany) at 4^o^C for 30 min. 

Subsequently, cell suspensions (up to 1×10^7^ cells/500 μl) were applied onto a MACS column in a magnetic field of a MACS separator, followed by washing the column with 500μl MACS separation buffer three times. While cells passed through the column, magnetically labeled CD146^+^ cells were mostly retained on the column and the unlabeled cells were eluted. The retained CD146^+^ cells were flushed out firmly and collected for further research. Trypan blue staining was performed to determine sorted cell viability and the CD146^+^ cells were cultured with complete DMEM/F12 prior to any assessments. 

**Preparations for karyotyping** **of isolated CD146** ^+^ **cells**

After isolation of CD146^+^ cells by MACS, the cells should be checked for chromosome number and banding pattern to determine if they were altered from the normal (expected) pattern. Karyotypic analysis (n=3) was performed according to routine methods, briefly, the CD146^+^ cells were harvested at 70-80% confluency (1×10^6^ cells) and treated in culture medium containing 0.15 ml of 50 µg/ml colchicine solution to each culture tube and followed by incubation at 37^o^C for 30 min (23). After 10 min centrifugation at 500×g, 2-3 ml of rewarmed 0.075 M potassium chloride (KCl) solution as a hypotonic buffer was added to the cell pellet at 37^o^C for 15 min. Then cell suspensions were gently fixed in 3:1 methanol-acetic acid and followed by overnight incubation at 4^o^C.

A small drop of cell suspension was applied onto the surface of a slide to spread and air-dried. Finally the slides were stained by Giemsa solution (Sigma, Munich, Germany) to perform cytogenetic analysis. Then the CD146^+^ cells were divided into two groups and they were cultured by different concentrations of SCF (0, 12.5, 25, 50 and 100 ng/ml) for 24 hr and 72 hr respectively.

**Proliferation** **of SCF treated cells by MTT assay**

For assessment of CD146^+^ cells proliferation following SCF, the tetrazolium compound MTT [3-(4,5-dimethylthiazol-2-yl)-2,5-diphenyltetrazolium bromide] was added to cultured cells. Briefly, cells were seeded into two different groups of wells at a density of 2×10^4^ cells/in 24 well, and then volumes of 100 μl of fresh medium supplemented with 10% serum were dispensed in triplicates with different concentrations of SCF including 0, 12.5, 25, 50 and 100 ng/ml. Then two groups of wells were incubated at 37^o^C in 95% humidified CO_2_ incubator (5% CO_2_ and 95% O_2_) for 24 hr and 72 hr respectively. After 24 hr and 72 hr of culture, 100 μL of 0.5 mg/ml MTT reagent (Sigma, Munich, Germany) was added to each well and the cells were incubated for 4 hr at 37^o^C. 

At the end of incubation period MTT is reduced by metabolically active cells, So insoluble purple formazan dye crystals were produced and deposited within the cells. After removing of MTT solution, 100 μL of dimethyl sulfoxide (DMSO) were added to each well that causes destructing cell membranes and solubilizing the crystals and then the absorbance was read using a spectrophotometer. The rate of tetrazolium reduction is directly proportional to the rate of cell proliferation (24). 

Finally, the optical density (OD) was measured at 570 nm using a microplate reader (Bio-Rad, Calofornia, USA). The average values from triplicate readings of SCF stimulated versus un-stimulated wells were determined and used to calculate the stimulation index (SI) as follow: SI=mean of OD values of mitogen stimulated wells/mean of OD values of un-stimulated wells (25).

**Statistical analysis**

Statistical analysis was performed using SPSS software (version 17.0, USA). The results were analyzed by one-way ANOVA and post-hoc Tukey's tests between the groups. P<0.05 was considered to be statistically significant. All data were described as Mean±SEM.

## Results

**The morphology of human cultured endometrial cells**

**Figure 1:**
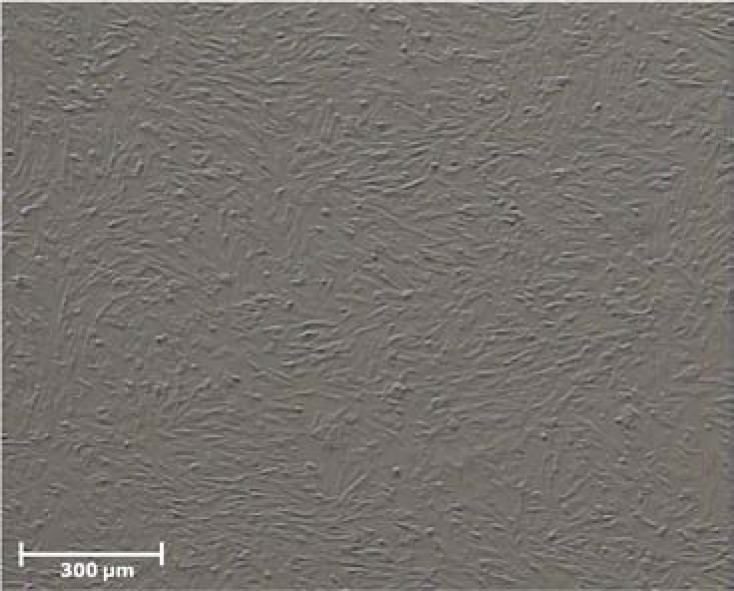
Phase-contrast photomicrograph of cultured human endometrial cells at passage four.

The figures of cultured cells at fourth passage showed uniformed cells. Cells usually appeared elongated and spindle-shape with round nuclei (Figure 1). 

**Purification of endometrial CD146 cells after MACS isolation**

**Figure 2:**
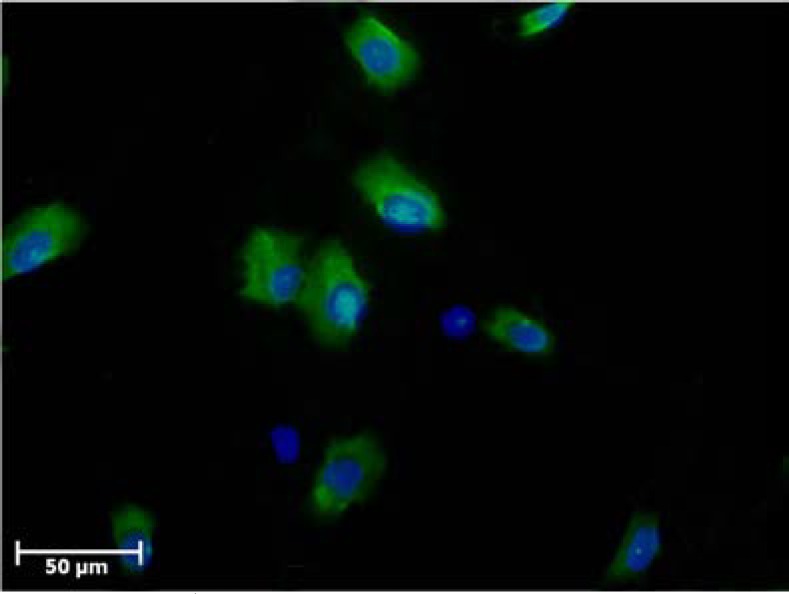
Immunofluorescence staining of CD146^+^ cells after isolation by MACS. Representative photomicrographs showing confirmation of isolated cells by MACS. The cells were labeled with FITC-conjugated secondary antibody and counterstained with DAPI (blue). Green colors show positive reaction for CD146 expression.

The survival rate of cells after MACS was 90±3.2% (n=6). The purification of isolated CD146^+^ cells by MACS was confirmed by immunocytochemistry under fluorescent microscope. The CD146^+^ cells per nucleated cells were 83±1.8% (n=6, Figure 2). 

**Karyotypic analysis**

**Figure 3:**
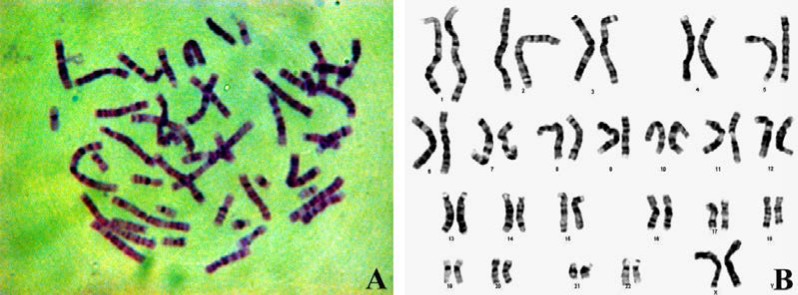
**The karyotyping results of** isolated CD146^+^ cells. They were assessed for lack of karyotypic abnormalities. Detailed analysis of cultured cells showed normal karyotypes containing 23 pairs of chromosomes. (B) Arrangement of the pairs of the chromosomes is shown.

Fifty metaphases were imaged and twenty cells were karyotyped with G-banding analysis. Normal karyotype of 46 chromosomes for isolated CD146^+^ cells was obtained (97±2.4%). Detailed analysis showed 22 autosomal pairs and two large X chromosomes (the inactive X may be very darkly stained). No abnormalities in quantity (aneuploidy, tetraploidy) or quality or other visible abnormalities were observed. This suggests that human EnMSCs are capable of large scale expansion without mutagenesis at the chromosomal level (Figure 3).

**Effect of SCF on cell proliferation**

**Figure 4:**
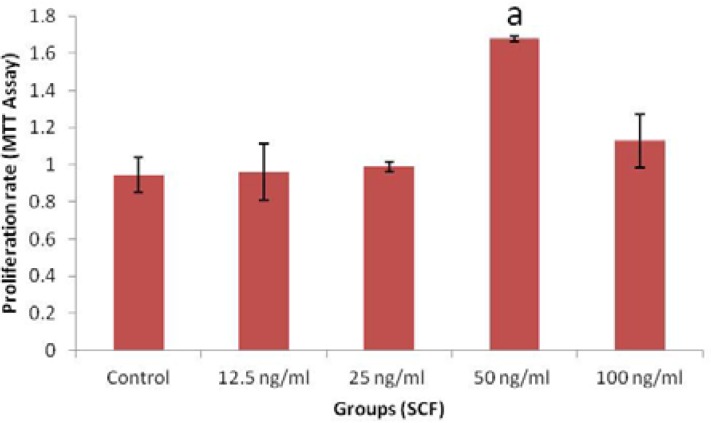
The effect of different concentrations of SCF on endometrial CD146^+^ cells proliferation. a: There was a significant difference between 50 ng/ml with other groups (p=0.01).

The proliferation rate of endometrial CD146^+^ in the presence of 0, 12.5, 25, 50 and 100 ng/ml SCF were 0.945±0.094, 0.962±0.151, 0.988±0.028, 1.679±0.012 and 1.129±0.145 respectively. There was a significant increase in EnMSC proliferation following in vitro treatment by 50 ng/ml than other concentrations of SCF (p=0.01, Figure 4). There was no significant difference between untreated and 12.5, 25, 100 ng/ml treated cells.

## Discussion

Results obtained in the present study for the first attempt revealed that human isolated endometrial cells which were treated by 50 ng/ml SCF exhibited a significant increase in proliferative response in comparison with other groups. Similarly, researchers revealed that 50 ng/ml is the optimal concentration of SCF for cell survival and proliferation of progenitor/stem cells (26). Moreover, it has shown the effects of SCF on neural crest cells growth, their proliferation rate and melanocyte differentiation in cultures treated with SCF (50 ng/ml) were significantly higher than the other concentrations (27).

The SCF is an important factor, ligand of the receptor tyrosine kinase C-Kit carried by different stem cells; primordial germ cells, hematopoietic stem cells and pre-melanocyte neural crest cells (28, 29). SCF is a cytokine that promotes hematopoietic stem cells self-renewal and binding to its receptor could stimulate hematopoietic proliferation and activate integrins on the cell surface to maintain the cell adhesion (30-32). SCF is responsible for expansion and differentiation of progenitors and acts primarily as a mitogenic factor promoting stem cell proliferation and it has a minor role in inhibition of apoptosis (26). 

A study focused on functions of SCF mediated through mTOR/p70S6 pathway. They have shown that mTOR, p70S6 kinases and their downstream signaling molecules 4EBP1 and S6 proteins are all activated by SCF in erythroid progenitors (26). So it is shown that SCF is one of the factors to initiate the protein translational machinery and activation of mTOR/p70S6 pathway leading to protein translation and cell proliferation (26). Therefore, the regulation of the protein translational mechanisms that is vital for cellular proliferation is controlled primarily by SCF in stem cells. 

In other point of view, karyotyping is a laboratory technique used to analyze chromosomes in order to look for any chromosomal anomaly which may occur during culture system. In this study the isolated human stromal CD146^+^ cells from in vitro culture of endometrial cells at fourth passage were shown a stable normal 46XX karyotype. In a similar investigation, Cervelló *et al* 2011 cultured side population cell lines of human endometrium for 12-15 passages (20 wks) and then cryopreserved. They demonstrated endometrial cell lines display normal 46XX karyotype and intermediate telomerase activity pattern (33). However there were some reports which showed during in vitro culture, the percent of normal karyotype stem cells were decreased (7, 33). Our observation in this part of study proved the efficiency of culture system.

## Conclusion

In conclusion, the present study suggests that SCF has positive effect in a dose-dependent manner on the proliferation and survival rate of EnMSCs which has important implications for biomedical sciences and applied cell studies in reproduction.

## Note

***This article extracted from Ph.D. thesis. (Mehri Fayazi)***
